# Lung Cancer Screening: Implementation Challenges and Health Equity Considerations For the Western Pacific Region

**DOI:** 10.1200/GO.22.00329

**Published:** 2023-02-07

**Authors:** Claire Nightingale, Claire Bavor, Emily Stone, Nicole M. Rankin

**Affiliations:** ^1^ Center for Health Policy, Melbourne School of Population and Global Health, University of Melbourne, Melbourne, Australia; ^2^ Department of Lung Transplantation and Thoracic Medicine, St Vincent's Hospital Sydney, Darlinghurst, Australia; ^3^ Faculty of Medicine, University of New South Wales, Kensington, Australia; ^4^ Sydney School of Public Health, The University of Sydney, Camperdown, Australia

## INTRODUCTION

The WHO Western Pacific Region (WPR) is home to 37 countries/areas and 1.9 billion people. It is economically, culturally, and linguistically diverse, containing some of the world's most populous countries such as China, to some of the least, such as Niue and Tuvalu. Its health systems must serve populations facing the impacts of climate change, from vast geographic areas to tiny island territories, as well as those with a history of colonization. These factors all pose challenges to the equitable provision of health services, including cancer screening.

**CONTEXT**

**Key Objective**
This article describes the challenges of implementing lung cancer screening (LCS) in the Western Pacific Region (WPR), outlining lessons learned from international trials and existing real-world programs, as well as successful strategies used in other cancer screening programs.
**Knowledge Generated**
The WPR has nearly half the global burden for lung cancer mortality, along with great diversity of health services and economic conditions. Future implementation of LCS must consider primary and secondary prevention programs, as existing tobacco strategies may be most effective in low- and middle-income countries where there is insufficient infrastructure to support LCS programs.
**Relevance**
To enable equitable implementation of LCS, strategies used in other clinical settings need to be tested across the WPR to address a lack of evidence. This includes adapting the strategies used in the six countries and two special administrative regions in the WPR that have experience of implementing LCS programs.

The WPR cancer burden is significant. In 2020, it bore 34.4% of all new cancer cases and 39.4% of all cancer-related deaths globally.^1^ Lung cancer was the most diagnosed cancer, accounting for 16.1% of all cases and almost a quarter of deaths (22.7%), the highest of any WHO-defined region.^1^ The WPR has 49.7% of the global burden for lung cancer mortality.^1^ There is substantial regional variation in lung cancer incidence because of varying rates of tobacco smoking and air quality, with age-standardized rates ranging from 6.5 per 100,000 people in Fiji to 42.2 per 100,000 people in New Caledonia.^1-3^ There is less variation in mortality as most cases are diagnosed as metastatic disease.^4^ Survival in high-income countries (HICs) may be better because of improved detection and access to treatment.^2^ Inequity is evident, with First Nations people in Australia and New Zealand experiencing higher incidence and mortality rates than non-Indigenous people.^5,6^

This review provides a background to lung cancer in the WPR, including the risk factors and primary and secondary prevention measures. The key considerations for lung cancer screening (LCS) are summarized, highlighting the implementation challenges encountered to date. The review considers broader issues of health equity, other public health priorities, and lessons learned from other cancer screening programs. We highlight the need for greater global advocacy to ensure that, if implemented, LCS is beneficial and not a driver of health inequities.

## METHODS

We ran online searches to find peer-reviewed and gray literature about LCS relevant to the WPR. This information was collated, reviewed and summarized to provide examples of relevant research and practice. We compiled lung cancer incidence and mortality data from the International Agency for Research on Cancer and current tobacco smoking, computed tomography (CT) scanner availability, workforce capacity data from WHO fact sheets, and reports for the WPR countries/areas reported. We sourced additional peer-reviewed and gray literature from PubMed and government websites/reports to summarize the status of other cancer screening programs (Data Supplement).

## RESULTS

### Risk Factors: Tobacco Smoking

Tobacco smoking accounts for approximately 85%-90% of lung cancer cases and two thirds of deaths globally.^4,7^ Incidence rates largely reflect the maturity of the tobacco epidemic, tending to be greater in HICs.^8^ However, this is shifting as most people who smoke tobacco now live in low-middle– and low-income countries (LMICs).^8^ Smoking prevalence across the WPR is high, especially among males, ranging from 15% to 16% in Australia and New Zealand to more than 50% in Papua New Guinea and the Solomon Islands.^9^ Smoking rates among females are lower, like in Vietnam (1.2%) and China (1.8%), with exceptions being Nauru (45.2%; Table 1).^8^ Although prevalence rates are decreasing across the WPR for males and females, the region is not on track to achieve the WHO 30% relative reduction in smoking rates.^10^ Although some countries have achieved marked reductions, such as Australia, where daily tobacco smoking rates have halved in the past three decades, there have been inconsistencies within communities. For instance, smoking rates among Aboriginal and Torres Strait Islander peoples (37.4%), and people living in remote Australia (19.6%) are substantially higher than in the broader population (11%).^11^

**TABLE 1:**
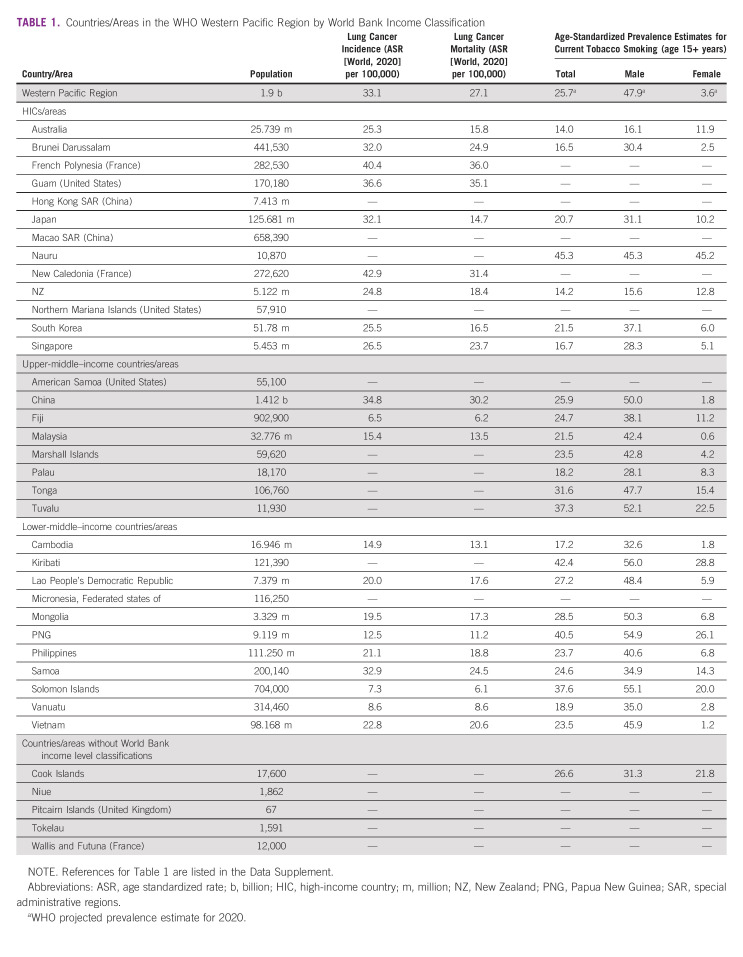
Countries/Areas in the WHO Western Pacific Region by World Bank Income Classification

### Risk Factors: Beyond Tobacco

Approximately 10%-15% of lung cancer cases in the WPR are detected in people who have never smoked.^12^ Tuberculosis is an important risk factor as some of the world's highest incidence rates occur in the Philippines and Papua New Guinea.^13^ Environmental and occupational exposures, family history, and genetic risks are significant contributors.^14,15^ Exposure to outdoor air pollution, including from particulate matter 2.5 (particles < 2.5 μm), accounts for 14.1% of lung cancer deaths globally.^16^ New evidence suggests that increasing levels of particulate matter 2.5 increases lung cancer risk in people with certain oncogenic mutations.^17^ WHO reports that 92% of the world's population lives in areas exceeding air-quality guidelines,^18^ including countries with increasing industrialization, population density, and vehicle emissions, such as China and Vietnam.^19^ Indoor air pollution from secondhand smoke, the use of solid fuels for cooking and heating, and radon in soil and water particularly affects women.^9^ For instance, one in five lung cancer cases in Chinese women is linked to secondhand smoke.^20^

Exposure to carcinogenic agents such as asbestos is linked to 62.7% of all occupation-attributable cancer deaths globally, the majority being lung cancer.^21^ Although asbestos use is largely eliminated in HICs and LMICs, there can be a 10- to 30-year lag between exposure and diagnosis.^22^ In 2016, Australasia and Asian HICs had the highest per-capita deaths from asbestos.^21^ Asbestos exposure in developing countries is less well quantified but may still be high in countries with large industry and manufacturing activity.^22,23^ Other agents commonly linked to cancer-related deaths in LMICs include diesel engine exhaust and silica.^21^

### Primary Prevention of Lung Cancer

Tobacco control is the most important approach to the primary prevention of lung cancer.^24^ The WHO Framework Convention on Tobacco Control is the most influential global plan for reducing the demand of tobacco products.^25^ The WPR is the only region to have all Member States party to the Convention, demonstrating a commitment to implementing effective interventions, including the WHO MPOWER (Monitoring, smokefree Policies, Offer help to cessation, health Warnings, Enforcing advertising bans, and Raising taxes) measures.^26^ Implementation of MPOWER measures varies across the WPR, with Australia, Brunei Darussalam, and New Zealand having some of the strongest policies.^8^ Twenty-four countries in the region have implemented at least one MPOWER measure at best-practice level.^27^ Implementation continues to improve, with the Cook Islands and Philippines joining the best-practice group for tobacco use cessation services and China for monitoring in 2020.^8^ However, WPR tobacco smoking rates are decreasing slowly in comparison with other regions.^10^ This likely reflects challenges in implementing cancer control policies and increasing use of electronic nicotine delivery devices.^27,28^

### Secondary Prevention: LCS

Large-scale international randomized controlled trials of LCS have demonstrated the clinical effectiveness of low-dose computed tomography (LDCT). Two landmark trials, the United States National Lung cancer Screening Trial (NLST), and the Netherlands/Belgium NELSON trial, reported a 20%-24% reduction in lung cancer mortality.^29,30^ The significant detection of early-stage disease (stage I and II) in both trials (57% and 67.9%, respectively)^29,30^ addresses the decades-old challenge of lung cancer being diagnosed at a late stage. This stage shift has been noted across the WPR in trials and pilot programs conducted in Japan, China, and Taiwan,^31^ and the K-LUCAS pilot in South Korea reported that 67% of diagnoses were early-stage.^32^ Similar findings are reported in real-world programs in the US Veterans Health Affairs Program (71% early-stage lung cancers)^33^ and five UK programs (81%).^90^

All screening programs have benefits and harms. Potential LDCT harms include radiation exposure, false positives, unnecessary procedures, overdiagnosis, and potential psychologic distress for participants.^34^ Innovations across international LDCT trials have shown harm reductions through improved detection and classification of lung nodules, including false positives (from 24% in NLST^29^ to 1.2% in NELSON trial)^29,30^ and unnecessary procedures for benign conditions (from 9% in NLST to 0% in NELSON trial).^35^ However, management protocols for diagnostic investigations and complications must be developed for the WPR, such as those described in the K-LUCAS pilot.^32^ Thus, although consensus in HICs that LCS benefits outweigh harms, further trials and pilot programs in the WPR are required, as is generating evidence about LCS cost-effectiveness.

Significant barriers to LCS uptake have been identified, including practical barriers (travel time and associated costs, work and/or career responsibilities), the impact of comorbidities, and emotional barriers (fear, shame, fatalism, avoidance, and low risk perception) inclusive of stigma.^36-39^ To enable equitable participation, LCS programs need to address age, sex differences, current and past smoking status, socioeconomic status, and geographical factors.^38^

### Components of LCS

The components of high-quality screening programs for optimal implementation have been recommended in HICs.^34,40^ Six countries and two special administrative regions in the WPR have experience of implementing LCS (Table 2). In this section, we outline these components and implementation strategies, drawing on LCS exemplars from the WPR. Successful strategies used in the WPR to implement other cancer screening programs are discussed later in this article.

**TABLE 2:**
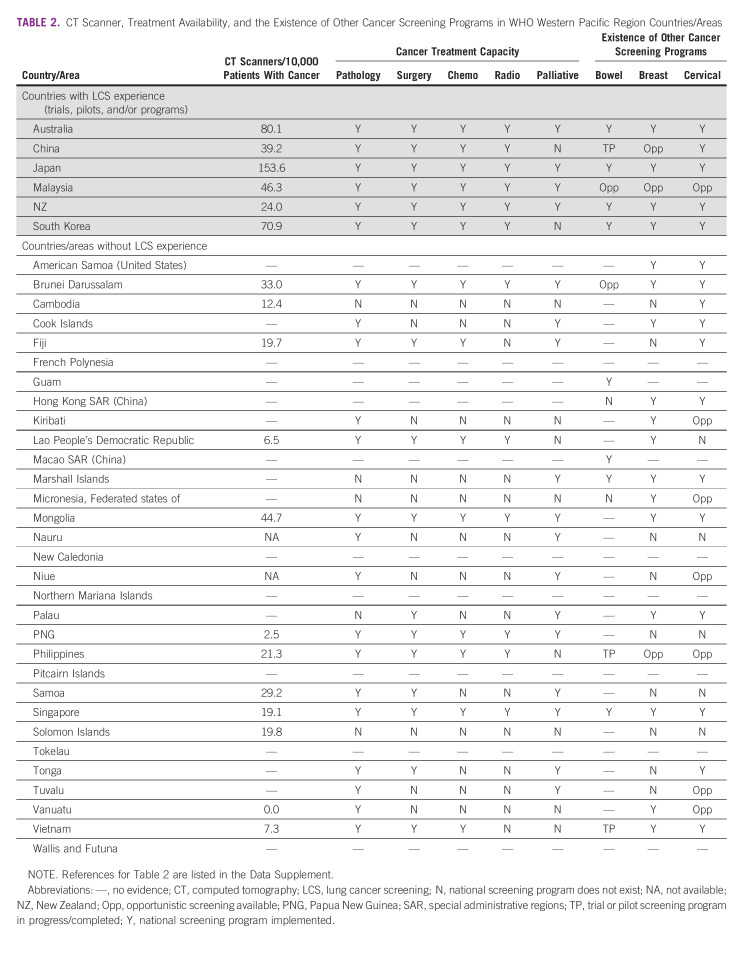
CT Scanner, Treatment Availability, and the Existence of Other Cancer Screening Programs in WHO Western Pacific Region Countries/Areas

#### Selecting the high-risk population.

LCS targets people at high risk in the population. Promotion and awareness raising is essential for increasing LCS knowledge. Common strategies include public awareness campaigns and behavioral interventions using online advertising and printed materials.^41,42^ There is little published evidence about the most appropriate strategies for the WPR. Recruitment strategies include mailing invitations, community outreach, and mass media.^43^ Strategies used within the WPR include offering LCS as part of an annual occupational health check^44^ and invitations from general practitioners.^45^ Most international trials report large nonresponse rates to invitations.^43^ For example, in Malaysia, a pilot LCS program was stopped early because of suboptimal recruitment likely due to participant refusal and a fear of diagnosis.^46,47^ In a Chinese LCS program that achieved a participation rate > 50%, people who had never smoked had higher education levels, a family history of lung cancer, and occupational exposure, and had higher rates of participation.^48^ Male participants have also been over-represented in international trials,^49^ and more evidence is needed about the optimal strategies to recruit female participants. A lack of centralized patient registration systems in most WPR countries precludes the systematic mailing of invitations, appointment reminders, and recalls after screening.

Eligibility criteria are typically determined on the basis of criteria of age and smoking history. Risk assessment tools are a more sophisticated way of selecting those at high risk. Inclusion for occupational or secondhand smoke exposure is not currently incorporated into pilot (eg, K-LUCAS) and proposed programs (eg, Australia).^50^ Asian trials investigating LCS for never-smokers are ongoing.^44,51^ Across the WPR, risk models are in development stages, and mapping the common causes of lung cancer is critical to developing appropriate risk assessment tools. A recent review highlighted an urgent need for external validation and model optimization for the Chinese population.^46^

#### Health care professional education.

Improving health care professional's awareness and knowledge of LCS including benefits and harms is another essential component.^52^ Primary care practitioners are the key workforce in many jurisdictions who will champion screening. Evidence from HICs shows that primary care practitioners are key in encouraging the participation of high-risk individuals.^53,54^ In most WPR settings, health care is delivered at multiple levels, with primary care, often nurse-led, typically provided at village/community-based level with referral to provincial and national tertiary settings when necessary. Future LCS implementation will require adaptation to health care settings in the WPR. Shared decision making is a mandatory component of LCS in other jurisdictions, but not yet a common practice in many WPR countries.^55-57^ The development and use of decision aids appropriate for the WPR requires further study. Finally, patient navigators may be appropriate in health systems across the WPR. To support LCS programs, health care professionals need education in optimal recruitment methods, the provision of smoking cessation support, communicating scan results, reading and reporting LDCT scans, and managing and investigating suspicious nodules and incidental findings. Health care professionals play an important role in reducing stigma in the health system and encouraging inclusivity of all people at high risk. It is likely that additional training to address stigma is needed.

#### Embedding smoking cessation interventions.

The integration of smoking cessation interventions into LCS programs maximizes participant benefits and cost-effectiveness. The most effective strategies for incorporating smoking cessation interventions at multiple points in the screening and assessment pathway are not yet identified.^58^ In the WPR, the effectiveness of mandatory cessation counseling with a physician was tested in the K-LUCAS pilot, which detected a 12.7% (*P* = .007) change in participant's willingness to quit smoking after screening.^59^ However, as 14% of LCS participants were recruited from smoking cessation services, they may have had a greater motivation to quit than the participants recruited via the National Screening Program (84%). In the now-implemented Korean National Lung Cancer Screening Program, there was broad support for the mandatory inclusion of in-person smoking cessation counseling, particularly from those eligible for screening (57.6%).^60^ Evidence in this area is rapidly changing. A microsimulation model of a nurse-led cessation intervention made LCS more cost-effective in the Chinese setting.^61^ Harnessing successful tobacco cessation initiatives outside LCS, such as the empowerment of community health workers to deliver interventions in LMICs, should be explored in the LCS setting.^62^ Efforts to support smoking cessation are supported at a policy level as countries in the WPR make progress toward achieving the best practice measures outlined in the MPOWER framework, notably, the increase in the number of countries that offer cost-covered nicotine replacement therapy, and have increased taxation and education campaigns.^24^

#### Physical infrastructure and human resources.

Before LCS implementation, WPR countries will need thorough screening and assessment pathways, agreed standards for LDCT scan delivery and results interpretation, and nodule management guidelines to reduce false positives and incidental findings.^34^ Access to an adequate number of CT machines with the technical capacity for monitored low-radiation doses appropriate for screening and sufficiently qualified and accredited radiologists and radiographers are essential. Australia and Japan have the greatest number of CT machines per capita.^48^ However, many WPR countries have few CT machines and limited numbers of appropriately skilled thoracic radiologists and radiographers (Table 2). Thoracic surgeons, clinical oncologists, and specialist oncology nurses are essential to the delivery of a LCS program. Robust quality assurance programs must be established alongside national LCS registers to monitor outcomes and enable research and continuous improvement.

#### Referral pathways, incidental findings, and coordination to treatment.

LCS programs require appropriate and accessible referral pathways and specialist capacity to treat people with identified cancers and manage incidental findings. The most common incidental findings include pulmonary, cardiovascular, and gastrointestinal comorbidities, many of which are clinically insignificant.^63^ Incidental findings that are clinically significant, such as coronary artery calcifications and osteoporosis, will require further management by primary care physicians. In cases where other cancers are detected, such as breast, adrenal, thyroid, and upper abdominal, referral for specialist clinical evaluation will be required.^64^ The detection of actionable findings is an advantage of the LDCT scan and presents a positive opportunity to implement standardized reporting recommendations for incidental findings and to minimize unnecessary workup of low-risk nodules. Cancer care and treatment in WPR is challenged by limited infrastructure and workforce capacity (Table 2). A recent WHO survey reported that many WPR countries do not have established referral systems for existing cancer screening programs.^65^ Challenges include poor access to essential medicines, palliative care and a reliance on out-of-country referrals for cancer treatment.^65^

Multidisciplinary teams (MDT) make treatment recommendations for people with screen-detected abnormalities and are important in reducing harms associated with overdiagnosis and unnecessary treatments.^66^ MDTs routinely guide decision making in Australia and New Zealand and are increasingly used in China, Japan, and South Korea.^67-69^ Most WPR programs offer LCS in metropolitan settings.^45^ It is critical to consider how people living in rural and remote areas will access screening and appropriate follow-up. This is particularly important, given workforce shortages of medical practitioners in primary and tertiary care outside metropolitan locations. The establishment of efficient and appropriate referral pathways, with the involvement of MDT, the use of standardized nodule management guidelines and leading to appropriately skilled thoracic surgeons, and oncology treatment, remains a challenge for every LCS program that has been implemented.

## DISCUSSION

Lessons learnt from LCS implementation should be coupled with learnings from decades of implementation experience of cancer screening and successful public health programs across the WPR. Encouraging innovation, continued workforce expansion, and aligning with and capitalizing on existing health strengthening initiatives will be important for the WPR in the future design and implementation of LCS. Global and regional WHO plans of high relevance to LCS mortality include two overarching regional initiatives^70,71^ that highlight whole of system health strengthening approaches.^71^ The WHO global strategy to accelerate the elimination of cervical cancer as a public health problem outlines a series of prevention and treatment targets. As the strategy actions are implemented, improvements in cancer registration and treatment infrastructure and capacity should harnessed to benefit all people diagnosed with any cancer.^72^

Across the WPR, 89% of countries have cervical screening programs, although only half (48%) have organized population-based programs and only 4% of countries having achieved ever in lifetime screening coverage of > 70%.^73^ Two thirds of countries have an implemented breast screening program, although about 25% adhere to best practice guidelines on screening age and interval.^73^ Few WPR countries have national bowel screening available (Table 2).

Local innovation and adaptation of screening programs to suit the resources and settings across the WPR has been successful. In both HIC and LMIC settings, cervical and breast cancer education, screening, and prevention programs have been delivered through community and occupational outreach models and dedicated screening days in countries including Hong Kong, Papua New Guinea, the Federated States of Micronesia, Cambodia, China, and Vietnam.^74-78^ Community health workers have delivered smoking cessation advice and clinical interventions but cite a need for more education to increase confidence in delivering cessation support and talking about cancer.^62,78-80^

Endorsement of primary care practitioners is an important facilitator to participation in screening programs, including for culturally and linguistically diverse people in HICs.^81^ Community education,^82^ multimedia, and SMS messaging approaches have demonstrated effectiveness in increasing knowledge and participation in cancer screening programs.^83,84^

Increasing global inequity is evident in cancer mortality. In HICs, primary prevention, including tobacco control and vaccination for hepatitis B and human papillomavirus, prevent cancers developing; greater resources to implement screening programs lead to more cases being diagnosed early with better treatment outcomes; and a greater availability and accessibility of treatment result in a higher proportion of cases being treated with curative intent, compared with LMICs. Using cervical cancer as an example, many HICs are on track to eliminate cervical cancer as a public health problem within our lifetime. However, nearly 90% of all cervical cancer deaths occur in LMICs; these deaths are preventable.

Participation barriers in cancer screening and poor access to treatment have a greater impact on marginalized and underserved populations.^80^ A lack of culturally safe services, language barriers, cultural beliefs about cancer, fatalistic attitudes, and the ongoing impacts of trauma and colonization contribute to First Nations People in countries such as Australia and New Zealand participating in national programs at lower rates compared with non-Indigenous people.^85^ Ethnic minorities and people living in lower socioeconomic areas participate at lower rates and experience worse cancer outcomes for screen preventable cancers.^80^ In the Pacific, identified barriers include a lack of knowledge and awareness among the general population, high-risk participants, and healthcare workers, limited access to health facilities, cultural beliefs, and cost.^47^ Financial barriers have been associated with delayed diagnosis or lower adherence to treatment in Vietnam,^80^ Australia, China,^86^ and New Zealand.^87^ Distance to services and spending a long time away from home are significant barriers. Financial toxicity after cancer treatment disproportionately affects people from rural areas, on low incomes, and from ethnic minorities.^86,87^ The critical importance of working directly with underserved populations to codesign recruitment materials and tailor models of service delivery to the population and setting has been an essential learning from programs to date and should be incorporated into any future LCS program design.^88,89^

In conclusion, lung cancer is a leading cause of global mortality. Evidence shows that LCS can reduce lung cancer mortality and detect disease at an early stage. However, substantial implementation challenges remain, which will continue to influence and shape the implementation of primary and secondary prevention programs across the WPR. To our knowledge, this is the first review to consider challenges across the entire WPR. Increasing health challenges associated with the impacts of climate change, aging populations, and an increased burden of noncommunicable diseases across the 37 WPR states create a substantial task in the prioritization of health spending. Systematic assessments of lung cancer burden, human and technical resource availability, and local cost-effectiveness will be needed across the WPR to inform health planning.

As evidence emerges, more LCS programs will be implemented and require a combination of primary and secondary prevention measures. It is critical that they are designed and delivered with equity considerations at their core. However, resource limitations and infrastructure challenges will mean that many LMICs will rely on tobacco control measures as the primary prevention strategy to reduce the impact of lung cancer. Thus, continued support for strengthening the implementation of the MPOWER measures in each country is critical. There is a need for greater global advocacy to ensure the innovation of LCS does not further drive cancer inequities.
